# Resettlement, mental health, and coping: a mixed methods survey with recently resettled refugee parents in Canada

**DOI:** 10.1186/s12889-023-15300-y

**Published:** 2023-02-23

**Authors:** Amanda Sim, Eve Puffer, Afreen Ahmad, Lina Hammad, Katholiki Georgiades

**Affiliations:** 1 Department of Psychiatry and Behavioural Neurosciences, The Offord Centre for Child Studies, McMaster University, Hamilton, ON Canada; 2 Department of Psychology and Neuroscience, Duke Global Health Institute, Duke University, Durham, NC USA

**Keywords:** Social determinants, Refugees, Parents, Resettlement, Post-migration, Daily stressors, Mental health, Coping, Resilience

## Abstract

**Background:**

Resettlement experiences of refugee parents are under-researched despite evidence indicating higher risk of poor mental health. The current study integrates family systems and social determinants of refugee mental health frameworks to examine: (1) Refugee parents’ experiences of resettlement stressors and mental health; (2) Perceived impacts of resettlement stressors on individual and family indicators of well-being; and (3) Refugee parents’ coping strategies and resources.

**Methods:**

The study draws on data from a mixed methods survey conducted with 40 Government-Assisted Refugee parents who had resettled to Hamilton, Canada within the past 4 years. Quantitative and qualitative data were analyzed separately and then integrated at the results stage using a weaving approach.

**Results:**

Results indicate significant exposure to economic and social stressors across multiple domains of daily life, as well as high levels of parental psychological distress. Parents drew linkages between resettlement stressors and negative mental health impacts that were compounded by intersecting risk factors of ill health, caregiving burden, single parenthood, and low levels of education and literacy. Most parents rated themselves as coping well or very well and described various coping strategies such as positive reframing, problem solving, planning, and turning to religion. Quantitative and qualitative findings indicate high frequency of positive parent-child interaction and low frequency of family conflict, and highlight the importance of family as a protective resource for coping with adversity. Exploratory regression analyses suggest that longer stay in Canada, poorer self-rated health, higher levels of resettlement stressors, and more conflict between adults in the household may be associated with greater psychological distress.

**Conclusion:**

Study findings highlight both the resilience of refugee parents and the psychological toll of navigating their families through a new and challenging environment. Policies and programs to provide comprehensive social and economic supports to refugees beyond the first one to two years after arrival are necessary to mitigate the mental health impacts of displacement over time and strengthen individual and family resilience. Such programs should include culturally responsive and family-based models of mental health care that acknowledge collective experiences and impacts of adversity, as well as harness family resources to overcome past and present challenges.

## Background

Global forced displacement has reached unprecedented levels as the number of refugees fleeing their countries due to armed conflict and persecution surpassed 20 million in 2021 [1]. The mental health burden of forced displacement is substantial, with recent meta-analyses indicating that up to 1 in 3 refugees in high-income countries has diagnosable depression or post-traumatic stress disorder [2]. In addition to potentially traumatic events in one’s country of origin and during flight, post-migration stressors exert a powerful influence on the mental health of refugees [3]. These include material stressors such as economic and housing insecurity as well as interpersonal stressors such as social isolation, discrimination, and concern about loved ones overseas – also conceptualized as social determinants of refugee mental health [4]. In addition, due to substandard living conditions and barriers to accessing healthcare, refugees often experience poorer physical health outcomes compared to the receiving country population [5]. Poor physical health status and reduced healthcare access have been associated with greater mental health difficulties among refugees [6]. A survey of Iraqi refugees resettled in the United States found that rating one’s health as fair or poor and having a commonly reported physical health condition (e.g., hypertension, diabetes) were each associated with more depressive symptoms [7].

There is now robust evidence that post-migration stressors increase the risk of mental disorder and generalized distress among forcibly displaced individuals, as well as intensify or prolong the mental health impacts of pre-migration trauma [8]. Furthermore, a longitudinal study with 2,399 adult refugees resettled in Australia found that associations between mental ill-health and resettlement stressors were gender-specific and time-varying. For example, positive associations between socioeconomic stressors and mental ill-health peaked around 1.5 years after resettlement for both genders while the association between difficulties adjusting to life in Australia and mental ill-health became more pronounced over time for male refugees in particular, peaking at around 3.5 years after resettlement [9]. These findings highlight the need to understand the relationship between resettlement stressors and mental health for different subgroups of refugees and at different stages of resettlement. Recently resettled refugee parents are a specific subgroup whose mental health is under-researched despite evidence indicating higher risk of poorer mental health outcomes among refugee and immigrant parents. The Longitudinal Survey of Immigrants to Canada, for instance, found that recent refugee and immigrant parents (i.e., arrived 6 months to 4 years ago) – particularly lone parents – had higher odds of reporting emotional problems compared to non-parents, and that refugee status, visible minority status, and female gender were associated with elevated risk [10].

Qualitative research with refugee parents provides some insight into the pathways through which pre- and post-migration experiences of trauma and adversity interact with acculturative stress and conflict to impair family functioning and exacerbate psychological distress [11, 12]. In a study with refugee new parents from Africa in Canada, participants described how lack of social support, shifting gender roles, and acculturative and parenting stress linked to parenting in a new culture contributed to marital conflict and strained family relationships [13]. Family conflict in turn has been found to significantly increase the risk of severe psychological distress among refugees in Australia and the United States [14, 15].

While there is growing evidence of the negative impacts of war and displacement on parental mental health and family processes [12], the ways in which refugee parents cope with and adapt to challenges and opportunities in their new environments are less understood [16]. Coping is generally defined as cognitive and behavioral efforts to manage external and/or internal demands that are appraised as taxing or exceeding one’s resources [17]. Theoretical frameworks have typically conceptualized coping as either problem-focused (i.e., efforts to manage or control the stressor) or emotion-focused (i.e., efforts to reduce or manage associated feelings of distress), while maladaptive coping responses may include avoidance (e.g., substance use) and mental or behavioral disengagement (i.e., giving up attempts to reduce or manage the stressor or resulting distress) [17]. Existing theoretical frameworks, however, may not adequately capture the range of cultural and contextual factors that influence how different conflict-affected and displaced populations cope. Qualitative research with Palestinian families, for instance, has shown how coping and resilience are distinctly rooted in resistance to occupation, return to cultural roots, and perseverance through daily adversities [18]. Associations between different coping strategies and mental health may also vary based on the nature or perception of the stressor. For example, a study with Syrian refugees in Turkey and Germany found that problem-focused coping – generally assumed to be adaptive – was associated with more anxiety and depressive symptoms among refugees on the Syria-Turkey border due to their ongoing proximity to the conflict, suggesting that certain forms of coping may be maladaptive in contexts where the stressor is perceived to be uncontrollable [19].

While family dysfunction and conflict are associated with poor mental health outcomes, family social support has consistently emerged as an important source of strength and resilience across studies with diverse refugee populations in different resettlement contexts [12, 20]. Qualitative research with Hmong, Syrian, and Somali refugees in the United States, for instance, found that family social support, sense of belonging in the family, family centrality, and ethnic community as an extension of the family played an integral role in coping with resettlement stressors and fostering positive adjustment [21]. These findings supporting the vital role of the family in influencing mental health, coping, and adjustment are consistent with a family systems perspective, which views the family as a dynamic system that is both impacted by stressful social contexts as well as a potential source of resilience [22]. Adopting a family systems approach can help to reframe research on refugee mental health, which has typically focused on individual-level risk and symptomology rather than the ways in which experiences of adversity, mental health, and resilience are rooted in the family context.

### Current study

The current study integrates the family systems and social determinants of refugee mental health frameworks described above to address the following research questions: (1) What are refugee parents’ experiences of resettlement stressors in Canada? (2) How do refugee parents perceive the relationship between resettlement stressors and individual and family indicators of mental health? (3) What strategies and resources do refugee parents use to cope with resettlement stressors and their impacts on mental health? We also conducted exploratory regression analyses to examine potential associations between participant demographic characteristics, health status, resettlement stressors, family conflict, and parental psychological distress.

The study draws on data from a mixed methods survey conducted in 2021 with 40 Government-Assisted Refugee parents who had resettled to Hamilton, Canada within the past 4 years. We focused the study on refugees resettled to Canada during this period given indications that mental health trajectories can vary significantly during the first few years of resettlement [9]. As many of the study participants had resettled to Canada during the COVID-19 pandemic, this also provided an opportunity to explore experiences of resettlement and mental health during the pandemic, although this was not a primary study objective. Using mixed methods allowed for triangulation of quantitative indicators of resettlement stressors, mental health, and coping with refugee parents’ own lived experience and understanding of how adversity and protective resources in the post-migration environment interact to influence individual and family outcomes [23]. The study aims to contribute to the growing literature on refugee parents’ mental health, coping, and resilience, with the goal of informing structural as well as individual and family-focused mental health interventions for this population.

## Methods

### Setting and participants

The study was conducted in collaboration with an organization that provides settlement services to newcomers, including Government-Assisted Refugees, in Hamilton, Ontario. Government-Assisted Refugees are typically referred for resettlement by the United Nations High Commissioner for Refugees (UNHCR) and receive settlement services (e.g., income support, housing assistance) funded by the Government of Canada for up to one year from the date of arrival [24]. Hamilton is the third largest city in Ontario with a population of approximately 582,000 [25]. Canada has emerged as a world leader in refugee resettlement, ranking first among the 26 countries offering opportunities for resettlement in 2018 and 2019 [26]. Hamilton ranks fifth nationally and second in Ontario for its proportion of foreign-born population at 24.1% [27]. Hamilton also has a proportionately larger refugee population than the national average, with approximately one-third of its foreign-born population entering Canada as refugees [27].

We focused the study on Arabic-speaking refugee parents given the large number of Syrian refugees who resettled to Canada since 2016 as part of the Canadian government’s humanitarian response [28]. Participant inclusion criteria were: (a) arrived in Canada as a Government-Assisted Refugee since 2018; (b) fluent in Arabic; and (c) primary caregiver to at least one child aged 4 to 17 years. Case workers from the service provider organization identified former and current clients who met the inclusion criteria and made initial contact by telephone to introduce the study and seek consent to share individuals’ contact information with the research team. Individuals who agreed to share their information were contacted by a member of the research team who read out the informed consent form in Arabic and documented oral consent. Informed consent procedures emphasized that all responses were confidential and that individuals’ decision to participate in the study would have no impact on their access to services.

### Data collection

Data collection was conducted by a team of 5 native Arabic-speaking interviewers who received training in research ethics, survey administration, and interview techniques from the first author prior to commencing data collection. All interviewers were female with lived experience of migration to Canada. Due to restrictions on in-person data collection during the COVID-19 pandemic, all interviews were conducted on a web conferencing platform such as WhatsApp or Zoom (80%) or on the telephone (20%). To address literacy-related barriers to participation, interviewers read out each survey question in Arabic and provided a pictorial depiction of the survey response options where appropriate. Interviewers entered participant responses directly into the Research Electronic Data Capture (REDCap) data management platform. Qualitative responses were documented verbatim in Arabic by the interviewers and translated into English by two certified translators. Information about Arabic language mental health services was provided at the end of the interview. All participants received a $25 gift card to thank them for their time and participation in the study.

### Measures

Existing Arabic translations of mental health measures were used where available. Survey items with no existing Arabic translation underwent translation and back-translation procedures by two certified translators and were further refined through pilot testing with 3 refugee parents who did not participate in the study.

*Resettlement stressors*. Participants rated the degree to which 15 resettlement-related stressors have been a problem for them or their family in the past 12 months (0 = *not a problem*, 1 = *a little bit of a problem*, 2 = *somewhat of a problem*, 3 = *a big problem*). The list of resettlement stressors was drawn from existing surveys including the Longitudinal Survey of Immigrants to Canada [30] and Building A New Life in Australia [31] and refined through discussions with the partner service provider organization. There was a high rate of missingness (> 10%) for 4 items (“looking for or finding a job,” “accessing childcare or child/youth services,” “accessing language or other courses,” “sponsoring/reuniting with family members”). Participants explained that these items were not applicable due to suspension of services during the COVID-19 pandemic. We created a sum score for resettlement stressors removing the items with more than 10% missingness and recoding missing values for the remaining items as 0.

*Parental and child health status*. Participants were asked to provide a global rating of their general health using a 5-point scale from 1 (*poor*) to 5 (*excellent*) (“In general, would you say your health is…”) [32]. Parents also rated the general health of a randomly selected child between the ages of 4 and 17 years old in their household using the same scale. Responses were recoded to be dichotomous (*poor*/*fair* = poor; *good*/*very*/*good*/*excellent* = good).

*Psychological distress*. The Kessler Psychological Distress Scale (K-6) is a six-item measure of psychological distress widely used to screen for depression and anxiety [33], including among refugee populations [34]. The K-6 includes six items about sadness, nervousness, restlessness, hopelessness, feeling everything is an effort, and worthlessness in the last 30 days. Responses were scored from 0 (*none of the time*) to 4 (*all of the time*) based on the last 30 days and summed to yield a minimum score of 0 and a maximum score of 24. We used the well-established cut point of ≥ 13 to indicate severe psychological distress [35]. This cut point, while not validated for Arabic-speaking populations specifically, has been used in studies with diverse refugee populations [14]. We used an existing Arabic translation of the K-6 available through the World Health Organization [36]. Internal consistency reliability was 0.81 in this study.

*Family functioning.* We adapted three items from the Canadian Health Survey on Children and Youth [32] and the Ontario Child Health Survey [37] to assess the frequency of positive parent-child interaction (“How often did you and your child talk, play or do something else enjoyable with each other?”), parent-child conflict (“How often did you and your child argue or fight?”), and inter-adult conflict (“How often were there arguments or fights between the adults in your family?”) during the previous 3 months. Response options were 0 = *never*, 1 = *less than once a month*, 2 = *once or twice a month*, 3 = *once a week*, 4 = *2–3 times a week*, 5 = *almost every day*.

*Self-rated coping.* A single item from the Building A New Life in Australia study [31] (“How well do you think you are coping?”) was used to assess self-rated coping. Responses were scored from 0 (*not at all*) to 3 (*very well*).

*Open-ended questions*. Participants were asked 6 open-ended questions during the survey. Questions were related to participants’ general resettlement experience (“Please tell me about your experience living in Canada”), resettlement stressors (“What have been the main difficulties or problems that cause the most distress, worry, or frustration to you and your family?”), the COVID-19 pandemic (“In your opinion, how connected are these difficulties or problems to the COVID-19 pandemic?”), impacts on parental mental health (“How have these difficulties affected you emotionally?”), impacts on family relationships (“How have these difficulties affected family relationships?”), and ways of coping (“How have you coped with these effects on your emotions?”). Responses were documented verbatim in Arabic by the interviewers and translated into English by two certified translators.

### Analysis

We used descriptive statistics including mean, standard deviation, frequency, and proportion to describe the demographic characteristics, resettlement stressors, and mental health of study participants. In addition, we ran exploratory regression models to examine associations between demographic characteristics, health status, resettlement stressors, and family conflict with the outcome of parental psychological distress, adjusting for age and gender, as well as independent associations of statistically significant variables with psychological distress. All quantitative analyses were performed in IBM SPSS Statistics version 26.0 [38]. We used the qualitative analysis software Dedoose [39] to conduct inductive thematic analysis of the qualitative survey responses [40]. Analytic steps included: (1) familiarizing ourselves with the data by reading and re-reading transcripts and noting initial ideas; (2) generating and applying initial codes to the data set (i.e. open coding); (3) refining initial codes to develop a coding framework; (4) applying the coding framework to the dataset including re-coding previously coded data where necessary; and (5) linking codes to generate, name and define themes related to the research objectives. Three coders (AS, AA, LH) independently coded data from 5 out of 40 participants. We discussed any discrepancies and revised the codebook as appropriate, which was then applied to the remaining data by two coders (AA, LH). We used an iterative, constant comparative approach to arrive at themes, after which we used a weaving approach to integrate qualitative and quantitative findings and present them thematically [41].

### Ethical and safety considerations

Ethics approval was obtained from the Hamilton Integrated Research Ethics Board (HiREB). All interviewers received training in confidentiality and informed consent prior to data collection, including how to respond to potential safety concerns. A safety protocol was developed for the study detailing the steps to follow in various risk of harm scenarios (e.g., suspected child maltreatment). There was one disclosure of a potential safety concern which was referred to a local child protection agency after discussion with the participant.

## Results

### Sample characteristics

Of the 48 parents who consented to be contacted about the study, 40 consented to participate and completed the survey, 3 declined, 2 were unreachable, 2 did not speak Arabic, and one had participated in pilot testing the measures and was not included in the study. Table 1 presents sample characteristics. The mean age was 43.3 years, 60% of participants were female, and 85% were married. Most participants (82.5%) were born in Syria and had a primary or secondary school education (77.5%). Over half (55%) arrived in Canada in 2018–2019 and the rest arrived in 2020–2021. More than half of the participants (57.5%) were unemployed and not currently seeking work due to illness, disability, being a full-time caregiver, or inability to speak English, and 90% relied on government assistance as their main source of income.

**Table 1:** Sample characteristics (n = 40)

*Characteristic*				
*Parent*				*Range/min-max*
	Age, *M* (*SD*)		43.26 (7.81)	30–66
	Female, *N* (%)		24 (60.0)	
	Country of birth, *N* (%)			
		Syria	33 (82.5)	
		Iraq	4 (10.0)	
		Sudan	3 (7.5)	
	Marital status, *N* (%)			
		Married	34 (85.0)	
		Separated/divorced	4 (10.0)	
		Widowed	2 (5.0)	
	Highest level of education, *N* (%)			
		No formal education	4 (10.0)	
		Primary school	16 (40.0)	
		Secondary school	15 (37.5)	
		Post-secondary certificate/diploma	3 (7.5)	
		Bachelor’s degree and above	2 (5.0)	
	Number of children under 18 years in same household, *M* (*SD*)		3.40 (1.82)	1–7
	Year of arrival in Canada, *N* (%)			
		2018	1 (2.5)	
		2019	21 (52.5)	
		2020	14 (35.0)	
		2021	3 (7.5)	
	Current employment status, *N* (%)			
		Not working and not seeking work	23 (57.5)	
		Full or part-time student	7 (17.5)	
		Unemployed and seeking work	5 (12.5)	
		Working full or part-time	4 (10.0)	
		Retired	1 (2.5)	
	Household main source of income, *N* (%)			
		Government assistance	36 (90.0)	
		Employment/self-employment income	2 (5.0)	
	Parental self-rated health status (% poor/fair)		65.0	
	Parental rating of child’s health status (% poor/fair)		28.0	
	Psychological distress, *M* (*SD*)		12.41 (5.66)	0–24
	Positive parent-child interaction^1^, *M* (*SD*)		4.37 (1.03)	0–5
	Parent-child conflict^1^, *M* (*SD*)		0.73 (1.45)	0–5
	Inter-adult conflict^1^, *M* (*SD*)		0.68 (1.25)	0–5
	Self-rated coping^2^, *M* (SD)		2.15 (0.71)	0–3

*M* = mean; *SD* = standard deviation.

^1^ Response options were 0 = never, 1 = less than once a month, 2 = once or twice a month, 3 = once a week, 4 = 2–3 times a week, 5 = almost every day.

^2^ Response options were 0 = not at all, 1 = not well, 2 = well, 3 = very well.

### High levels of resettlement stressors across multiple domains of daily life

Resettlement stressors that were most highly endorsed by parents were: (1) concern for the safety and well-being of loved ones outside Canada; (2) obtaining permanent, affordable or good quality housing; (3) sponsoring or reuniting with family members outside Canada; (4) paying back the Government immigration loan (i.e., loans provided by the Government of Canada to defray transportation and other costs of establishing residence in Canada); and (5) paying bills, rent or transportation (Table 2). The majority of participants (57.5–77.5%) rated these resettlement stressors as “a big problem.” A substantial proportion (37.5–79.3%) of participants also rated 8 other resettlement challenges (employment, access to child and youth services, communication in English, access to benefits and services, food insecurity, access to language or other educational courses, making friends, access to healthcare) as “somewhat” or a “big problem,” indicating high levels of resettlement-related stressors across multiple domains of daily life.

**Table 2:** Resettlement stressors

	*N*		*M (SD)* ^1^
Concern about loved ones overseas	40	2.70 (0.61)	
Permanent, affordable or good quality housing	39	2.69 (0.66)	
Sponsoring/reuniting with family members	33	2.67 (0.85)	
Paying immigration loan	38	2.39 (1.03)	
Paying bills, rent or transportation	40	2.30 (1.02)	
Looking for or finding a job	29	2.14 (0.99)	
Accessing childcare or child/youth services	29	2.00 (1.23)	
Communicating in English for essential tasks	39	1.85 (1.27)	
Understanding/accessing benefits and services	39	1.72 (1.12)	
Affording enough or good quality food	40	1.55 (1.01)	
Accessing language or other courses	35	1.34 (1.33)	
Making friends or getting to know community	40	1.20 (1.20)	
Accessing healthcare, medication, or medical equipment	40	1.18 (1.15)	
Communicating with loved ones overseas	40	0.53 (1.06)	
Feeling discriminated against	39	0.23 (0.67)	

^1^ Rated on a scale of 0 = *not a problem*, 1 = *a little bit of a problem*, 2 = *somewhat of a problem*, 3 = *a big problem*.

Qualitative findings also highlighted the wide-ranging and debilitating nature of resettlement stressors experienced by refugee parents in the study. In particular, difficulties with learning and communicating in English were described by almost all parents as having pervasive negative impacts on their ability to perform essential tasks such as accessing healthcare, seeking employment, navigating government benefits, and forming social connections. A 43-year-old mother of 6 from Syria described how her family’s lack of English language skills affected her children’s healthcare, posed a barrier to achieving independence, and contributed to mental distress:

*We don’t speak the language, it’s been a year and we still don’t speak the language and cannot manage our affairs. Our case worker might leave us and I don’t know if he will stay with us. I am anxious and worried, especially because of my sick children and their appointments with the hospital and the doctors… I don’t know what will happen.* (134P)

In addition, all refugee parents described how the high cost of living in Hamilton, inadequate income support from the government, and difficulty obtaining employment resulted in intense financial hardship and pressure. For almost half the parents, the lack of affordable and safe housing was a major challenge that affected their and their families’ physical and mental well-being. A 36-year-old mother of 5 from Iraq described feeling alone and helpless in her attempts to improve their dire housing situation, particularly as her lack of English proficiency and unfamiliarity with the social services system limited her capacity to advocate for herself and her family:

*Our whole house is full of mice…my baby daughter is less than one and a half years old and she ate the mouse droppings and got sick immediately with vomiting and diarrhea…If there is only someone who would help me…I cry and I ask but no one is willing to help me. I even beg. If they would just increase our allowance by a little bit then we would be able to move, or if they can give us a rent subsidy…It affected us, our psychological state is not good, we have not been at ease since we came…We don’t speak the language and no one is helping us…the children’s psychological state is bad and they are afraid of everything…all of them fear the mice wherever they go. I just want to leave this apartment, nobody is helping me. I spoke to welfare, they didn’t help me. They brought me a lawyer but we are afraid they will make problems for us or write reports on us and then not give us an apartment.* (114P)

### High levels of parental distress compounded by intersecting stressors and risk factors

Both quantitative and qualitative results indicate extremely high levels of psychological distress among refugee parents in the study. Half of the parents screened positive for severe psychological distress using a cut point of ≥ 13 on the Kessler-6 [35]. In open-ended responses, parents commonly used the Arabic term *nafsiah* (نفسية) – translated as “psyche” or “psychological state” – to describe their mental health as “tired,” “poor,” or “low.” Parents also described mental health-related concerns such as insomnia, fatigue, lack of appetite, fear, anxiety, depression, inability to concentrate, and constant worry or pressure. Several parents described a sense of regression or “starting from zero” that was further exacerbated by the widespread and prolonged disruptions to essential settlement services such as English language classes during the COVID-19 pandemic.

Most parents attributed their distress to current resettlement-related stressors with only a few mentioning ongoing psychological impacts of past exposure to war trauma. The daily stressors of language barriers, poor housing, family separation, inadequate income, and lack of timely access to healthcare were described as having an intersecting and compounding effect on parents’ mental health. These effects appeared to be particularly pronounced for single parents and those who were separated from family members, for whom lack of social support compounded their sense of isolation and helplessness. In the following quotation, a widowed mother of two from Syria explained how single parenthood and illiteracy intersected to compound the difficulties of adapting to her new environment, which in turn contributed to intense psychological distress:

*Praise be to Allah, I am suffering. I am alone and responsible for the house and I have children. Everything is difficult. I don’t read or write. I go on the bus and don’t know where I need to get off. I am responsible for every single thing…Everything I need to do or to get is strenuous, I am very tired…I constantly get lost, I am asked to do everything by myself. Yesterday I had a breakdown. People make fun of me because I don’t know anything…If I had studied Arabic, I would have been able to learn English by translating. But my situation is very difficult…every day I cry 4–5 times.* (112P)

Parents also described their own and their family members’ poor health status and associated caregiving responsibilities as having a significant mental health burden. Parents’ ratings of their own and their child’s health indicated generally poor health status, with 65% of parents rating their own health and 28% rating their child’s health as poor or fair. In open-ended responses, approximately half of the parents described health conditions such as high blood pressure, impaired vision, depression, and disabilities resulting from pre-resettlement injuries that impaired their own or their spouse’s daily functioning. Several parents noted how the long wait times for receiving healthcare added to their distress about their own or their family member’s health condition and prevented them from pursuing employment or educational opportunities. In open-ended responses, parents also described the physical and psychological burden of caring for children with special needs including physical disabilities such as deafness, chronic health conditions such as asthma, and developmental disorders such as autism. As noted by this 44-year-old father of six from Syria, the burden of poor health and caregiving intersected with other stressors including unemployment and low income to trap families in a cycle of economic and social disadvantage:

*Three of my children have a disability and they take all my time to care for them and take them to school. There is no way to improve my financial standing or find a job because I have to always drop them off and pick them up and they don’t go full-time to school, so I can’t get a full-time job.* (124P)

For many refugee parents, the COVID-19 pandemic and related lockdowns created additional barriers to adaptation by reducing opportunities for social interaction, language acquisition, and learning the cultural and societal norms of their new environment. The tension and family conflict arising from being in lockdown were further intensified for some families due to dashed hopes and unmet expectations of their resettlement experience, as explained by this 35-year-old mother of four from Syria:

*They* (children) *have more demands and they fight with each other at home because they are at home all the time. They blame us, why did you bring us here? I had promised them that when we go to Canada we will do so much and have fun and until today they blame me that in the summer they didn’t go to the pool and didn’t go out to have fun except in the park near our house.* (145P)

Some parents, however, noted that the difficulties of settling in Canada predated the pandemic or persisted even after lockdowns and other public health measures were lifted.

Exploratory regression analyses indicate that longer stay in Canada (i.e., arriving in 2018–2019 vs. 2020–2021), poorer self-rated health, higher levels of resettlement stressors, and more conflict between adults in the household were associated with greater psychological distress (Table 3). With the exception of length of stay in Canada, all other variables remained independently associated with higher levels of psychological distress in the fully-adjusted model.

**Table 3:** Basic- and fully-adjusted associations between demographic, health, resettlement stressors, and family conflict variables and parental psychological distress (n = 40)

Basic-adjusted model^a^			
		*b* (SE)	*p*
*Demographics*			
	Age	0.017 (0.114)	0.882
	Gender (female)^b^	3.287 (1.771)	0.071
	Country of birth (Iraq or Sudan)^c^	2.911 (2.283)	0.211
	Highest level of education	− .541 (0.873)	0.539
	Marital status (widowed/divorced)^d^	3.156 (2.589)	0.231
	Number of children in household	− 0.027 (0.496)	0.957
	Recency (arrived 2018–2019)^e^	4.717 (1.616)	0.006
	Employment status (employed/student)^f^	-1.838 (1.903)	0.341
*Health status*			
	Self-rated health (poor/fair)	4.652 (1.944)	0.022
	Parent-rated child health (poor/fair)	2.986 (1.861)	0.118
Resettlement stressors^g^		0.335 (0.120)	0.009
*Family conflict*			
	Parent-child conflict	− 0.080 (0.600)	0.894
	Inter-adult conflict	1.960 (0.654)	0.005
**Fully-adjusted model** ^**h**^			
		*b* (SE)	*p*
Age		− 0.058 (0.095)	0.544
Gender		0.805 (1.319)	0.546
Recency (arrived 2018–2019)		2.254 (1.343)	0.104
Self-rated health (poor/fair)		4.356 (1.478)	0.006
Resettlement stressors		0.306 (0.102)	0.005
Inter-adult conflict		2.005 (0.533)	< 0.001

^a^ Adjusted for age and gender

^b^ Reference category is male

^c^ Reference category is Syria; Iraq and Sudan were grouped together given the small *n*

^d^ Reference category is married

^e^ Reference category is arrived 2020–2021

^f^ Reference category is unemployed/not working/retired

^g^ Sum score of resettlement stressor ratings with missing values coded as 0, not including 4 items with > 10% missingness

^h^ Includes only variables with *p-*value ≤ 0.05 in basic-adjusted model and age and gender

### “We are fighters:” Ways of coping and family social support as a source of resilience

Despite overall high levels of resettlement stressors and psychological distress, 55% of parents reported that they were coping “well” and 30% reported that they were coping “very well.” Open-ended responses described several different strategies that parents used to cope with daily stressors and resulting feelings of distress including support seeking, problem solving, and religious coping. Some parents, such as this 37-year-old father of three from Syria, normalized resettlement challenges as “regular difficulties that are overcome” and described how their situation had improved over time, particularly after accessing services:

*Life is good here. It was difficult in the beginning when we first came especially because of the English language and adapting to a new society. But thankfully things are better now. We studied the language a little bit and things have started to settle.* (129P)

Several parents, such as this 43-year-old Syrian mother of two, discussed using coping strategies including planning, staying positive, and having hopes for their own and their children’s future: “Thank God my ambitions increased. I have to continue my studies and do something for my future. The doors are opening for me.” (127P) Some parents also described using cognitive restructuring strategies such as comparing their current circumstances to adversities they had experienced in the past and reminding themselves that they were accustomed to hardship and had survived worse conditions. This 32-year-old mother of two from Syria described how she engaged in both problem-focused coping and positive reframing to deal with the issue of bed bugs in her home: “I started to buy and spray the house myself, and I remind myself that the coming days will definitely be better, and that this is not the first time I start from square one.” (111P)

Cultivating acceptance and patience by “leaving it in God’s hands” was another common coping strategy. Many parents noted that “life has to go on” and that “depending on God” was often their only way to cope with hardship, particularly when problems were perceived to be out of their control. As explained by this 46-year-old mother of five from Syria, “God destined this fate for us” (135P), suggesting that for some parents, the notion of surrendering to God’s will and entrusting their fate to God was an important coping mechanism. Support from service providers such as settlement workers was also mentioned by some parents as a coping resource, although the level of satisfaction with services varied widely. While some parents described feeling even more isolated, let down, and abandoned when they were not able to receive the assistance they needed, the following quotation from a 50-year-old mother of one from Iraq illustrates how emotional and instrumental support from service providers – in this instance, a school-based settlement worker – can have significant positive impacts on parental and child well-being:

*Last year there was a lady at school who was like a liaison between families and the school who would help me a lot and cared for him like her own son. She gave us a lot of comfort and would worry about my son and would give us advice on what he should and shouldn’t do. She would guide me and would talk to him in her special way and would give him advice for his future.* (109P)

Notably, most study participants did not describe adverse impacts of resettlement stressors or parental psychological distress on family functioning and well-being. On average, parents reported frequent (i.e., 2–3 times a week) positive parent-child interactions and limited (i.e., less than once a month) parent-child and inter-adult conflict. Qualitative responses were generally consistent with most parents describing family relationships and interactions characterized by mutual respect and understanding, emotional support, patience, positive communication and conflict resolution, and a sense of friendship between parents and children. This 47-year-old Syrian father of three explained how he and his wife made an effort to maintain family harmony in spite of external pressures: “We try not to get on each other’s nerves, my wife and I, because our circumstances and situation are already difficult.” (129P) Some parents specifically noted that emotional support from family members was an important source of resilience that enabled them to cope with daily adversity. A 42-year-old Syrian mother of two said: “As long as the family deals with any matters with love and communication, there will be no problems.” (127P) For a few parents such as this 35-year-old mother of four from Syria, however, the stressors of resettling to Canada during the COVID-19 pandemic interacted with her partner’s pre-existing mental health condition, resulting in increased marital conflict and psychological distress:

*We fight with each other over the simplest things. This is the worst thing, we blame each other and feel a lot of pressure and a lot of anxiety. My husband was already depressed and it increased, now he is going to therapy sessions.* (145P)

Finally, several parents described keeping everything “bottled up” and not doing anything to cope or not knowing what to do to cope. The inability to improve their stressful circumstances resulted in a sense of helplessness and powerlessness, as explained by this 36-year-old mother of five from Iraq: “We try to make ourselves stronger but we can’t do anything, we can’t help each other and we have nothing to make us stronger.” (114P)

## Discussion

This study used data from a mixed methods survey with 40 recently resettled refugee parents in Canada to examine their experiences of resettlement stressors, mental health, and coping strategies and resources. Consistent with research with refugees in Canada [42], the United Kingdom [43], and Australia [14], our study found significant exposure to economic and social stressors across multiple domains of daily life, as well as high levels of psychological distress as assessed by a standardized mental health instrument. In open-ended responses, participants drew linkages between these stressors and negative mental health impacts that were compounded by intersecting risk factors of ill health, caregiving burden, single parenthood, and low levels of education and literacy. Results from exploratory regression analyses were broadly consistent with qualitative findings, indicating that poor parental health, exposure to resettlement stressors, and higher levels of inter-adult family conflict were associated with greater psychological distress. Levels of psychological distress appeared to increase with length of stay in Canada, highlighting the urgent need for prevention and early intervention. Overall, both qualitative and quantitative results support a social determinants of mental health framework positing that refugees are at greater risk of poor mental health due to adverse post-migration social and economic conditions [4, 42].

Despite significant exposure to resettlement stressors and psychological distress, the majority of refugee parents in the study rated themselves as coping well or very well. In open-ended responses, parents described various coping strategies such as positive reframing, problem solving, planning, and turning to religion that generally align with core problem-focused and emotion-focused coping domains identified in the literature [44]. Consistent with existing literature on mental health and coping among the Syrian population, parents’ conceptualization of God and fate appeared instrumental to their ability to understand, accept, and persevere despite overwhelming and unrelenting hardships [45]. Several parents, however, expressed a sense of helplessness and despair regarding the perceived uncontrollable nature of their situation, highlighting the need for further research on culturally informed cognitive and behavioral coping strategies that can be adaptive in conditions of pervasive stress.

Contrary to existing research on the negative impacts of forced displacement on parenting and other family processes [11, 46], refugee parents in this study reported high frequency of positive parent-child interaction and low frequency of family conflict on average. Participants’ open-ended responses highlight the importance of family as a source of strength and resilience, consistent with a family resilience framework which focuses on the capacity of the family system to withstand and rebound from stressful life challenges [22]. Qualitative research with recently resettled Syrian refugee parents in the Netherlands found similar evidence of more positive parenting and parent-child interactions despite significant resettlement and acculturative stress [12]. This emerging research suggests that displacement and resettlement-related impacts on family processes may not be uniformly negative and highlights the potential of the family system as a protective resource for refugee mental health. However, while levels of family conflict were generally low in our sample, results from exploratory regression and qualitative analyses suggest that inter-adult conflict as a specific type of family dysfunction may be an important risk factor for refugee parents’ mental health.

### Strengths and limitations

Strengths of the study include the collection and integration of quantitative and qualitative data from an under-researched population of recently resettled refugee parents, which helped to triangulate findings and provide a more comprehensive and multi-dimensional understanding of participants’ lived experiences. Given the small sample size, regression analyses were exploratory and results should be interpreted with caution. All study participants were Government-Assisted Refugees and over 80% were from Syria. Hence, the resettlement and mental health experiences of refugee parents from other ethnocultural backgrounds or who arrived in Canada as privately-sponsored refugees or refugee claimants may be different. Data collection took place during the COVID-19 pandemic, which presented a unique opportunity to examine the experience of resettlement in the context of unprecedented societal and health and social service system disruptions. Study findings should be interpreted within the specific context of the COVID-19 pandemic given challenges in disentangling pandemic-related from resettlement-related stressors. Finally, the survey included only one item to assess global self-rated coping. Participants’ open-ended responses suggested a range of coping strategies and resources, including several that were grounded in participants’ cultural and religious background. Further culturally-informed research on coping is needed to understand the relationship between different ways of coping and mental health among diverse refugee groups, in order to inform interventions to promote adaptive coping and reduce psychological distress.

### Implications

Overall, findings from this study highlight both the resilience of refugee parents and the psychological toll of navigating their families through a new and challenging environment. Our study underscores the urgent need to address structural and social determinants of refugee mental health, particularly in the first few years after resettlement when initial hope and optimism can quickly give way to despair and hopelessness without adequate supports [42, 47]. Further research on the longitudinal mental health impacts of resettlement stressors, parental health problems, and intimate partner conflict, in particular, is needed to identify targets for intervention. Canada’s commitment to refugee resettlement, while laudable, coincides with escalating cost of living and housing prices, particularly around the Greater Toronto and Hamilton Area where the majority of refugees in Canada settle [48]. Policies and programs to provide comprehensive social and economic supports to refugees beyond the first one to two years after arrival are necessary to mitigate the mental health impacts of displacement over time and strengthen individual and family resilience. Such programs should include culturally responsive and family-based models of mental health care that acknowledge collective experiences and impacts of adversity, as well as harness family resources to overcome past and present challenges [49, 50].

## Data Availability

The data that support the findings of this study are not publicly available due to inclusion of information that could compromise participant privacy but are available from the corresponding author on reasonable request.
